# 

**Published:** 1920-12-04

**Authors:** 


### Matrons' and Sisters' Appointments.

STIRLING DISTRICT ASYLUM, LARBERT, N.B.—Miss Phœbe G. Yeats has been appointed assistant matron. She was trained at Aberdeen Royal Infirmary.

FEVER HOSPITAL, DARLINGTON.—Miss F. Kinnear, A.R.R.C., has been appointed senior sister and deputy-matron. She was trained at the City Hospital, Edinburgh, and the Salop Infirmary, Shrewsbury, and has since been staff nurse at the West End Hospital, Welbeck Street, W. She was also night sister and ward sister at the Fever Hospital, Darlington, sister at Crook Isolation Hospital, and staff nurse and sister for four and a-half years in Queen Alexandra's Imperial Military Nursing Service Reserve.

CHILDREN'S HOSPITAL, EAST END BRANCH, SHEFFIELD.—Miss A. Vickers has been appointed sister-in-charge. She was trained at the Royal Hospital, Sheffield, has been sister at the Port Sunlight Hospital, at Bristol Eye Hospital, and at the Northern Hospital, Manchester. She served at home and abroad in the Territorial Force Nursing Service, and has been matron of the American Red Cross Babies' Hospital, Hull.

UNIVERSITY COLLEGE HOSPITAL, W.C.—Miss Aileen Moore has been appointed *x*-ray sister. She was trained at the Devon and Exeter Hospital, has served as sister in the Q.A.I.M.N.S.R. and in the *x*-ray department of the West London Hospital. She holds the certificate in massage and medical electricity of the (now) Chartered Society of Massage and Medical Gymnastics.

METROPOLITAN HOSPITAL, KINGSLAND ROAD, E.—Miss Lois Irene Newman has been appointed assistant matron. She was trained at the London Hospital, where she was later sister. She has also been sister-in-charge of the Military Hospital, Newlands Corner, Guildford, and assistant matron and acting matron of the Mines Hospital, Rio Tinto, Spain.

CHRISTIE HOSPITAL, MANCHESTER.—Miss Alice Walker has been appointed matron. She was trained at the Sheffield Royal Hospital, was sister T.F.N.S. at the 3rd Northern General Hospital, Sheffield, and later on active service; she has been matron of the Military Hospital, Darwen, and night sister and housekeeping sister at the Royal Waterloo Hospital for Children, London.

CITY OF WESTMINSTER UNION INFIRMARY, FULHAM ROAD.—Miss Alice H. Rawlins has been promoted to be night sister from ward sister. She was trained at the Kensington Infirmary, where she was later ward and night sister, and took her fever training at Ham Green Hospital, Bristol. She served for some time in the T.F.N.S. at the 3rd London General Hospital, Wandsworth.

BECKETT HOSPITAL, BARNSLEY.—Miss M. P. Browne has been appointed sister-tutor and theatre sister. She was trained at the Sheffield Royal Hospital and the Ruchill Fever Hospital, Glasgow. She has held the appointments of theatre sister, home sister, and assistant matron at her general training-school.

KENSINGTON AND FULHAM GENERAL HOSPITAL.—Miss Theresa M. Whittingham has been appointed matron. She was trained at Guy's Hospital, where she was sister for eight years, and later became matron of a Government hospital in Jamaica. More recently she has been sister-in-charge of an officers' hostel at Hampstead, and has since been engaged in private nursing.

DURHAM COUNTY AND SUNDERLAND EYE INFIRMARY.—Miss L. M. Crosbie has been appointed sister. She was trained at the Royal Infirmary, Manchester, and the Birmingham and Midland Eye Hospital, at which latter hospital she was later sister.

WEST LONDON HOSPITAL, HAMMERSMITH.—Miss M. K. Coggins has been appointed matron. She was trained at St. George's Hospital, where she was later sister, and has been home sister at Warneford Hospital, Leamington, assistant matron at the Sheffield Royal Infirmary, and matron of the Royal Infirmary, Wigan.

